# Pediatric Mild Asthma: Bridging Current Gaps to Improve Future Care

**DOI:** 10.1002/ppul.71800

**Published:** 2026-08-21

**Authors:** Francesca Santamaria, Cristina Fontanella, Melissa Borrelli

**Affiliations:** ^1^ Department of Translational Medical Sciences, Pediatric Pulmonology Unit Federico II University Naples Italy

**Keywords:** adolescents, asthma, children, mild asthma, wheezing

## Abstract

**Background:**

Pediatric mild asthma is considered a benign condition, yet it accounts for a substantial proportion of morbidity. Characterization and management of children or adolescents with mild asthma are less explored than in adults, particularly due to its heterogeneous nature and variable course. Current definitions of mild asthma in pediatric populations vary across international guidelines or reports, limiting standardization. Although knowledge of asthma phenotypes and endotypes, and risk biomarkers has progressed notably, it is incomplete in the different pediatric age groups exhibiting mild or intermittent symptoms. Moreover, differing treatment recommendations may lead to uncertainty in clinical practice on whether using short‐acting β_2_‐agonist monotherapy or intermittent or daily inhaled corticosteroids or the regimen with inhaled corticosteroids plus bronchodilators combined in single‐inhalers.

**Methods:**

In this narrative review, we integrated evidence and recommendations from major asthma guidelines and reports complemented by selected insights from recent literature not addressed in these sources.

**Results:**

We summarized the current knowledge of pediatric mild asthma, risk factors for exacerbation, treatment recommendations, and highlighted gaps in clinical practice or questions still unanswered.

**Conclusions:**

In children and adolescents, asthma may seem only apparently mild, mainly due to overlooked or poorly controlled exacerbations. A more unified approach would improve clinical outcomes and reduce its global burden. Priorities include a standardized definition of the mild asthma phenotype in children and adolescents, a detailed examination of risk factors as early predictors of poor outcomes and of the limited access to medications and healthcare resources in low‐income countries. Incorporating a broader global health perspective would provide a more comprehensive understanding of the factors influencing the care of children with mild asthma worldwide.

## 1 Introduction

Most patients with asthma seen in primary care have intermittent symptoms that do not consistently affect their daily lives and are therefore classified as having mild asthma (MA) [1]. Despite this, the burden of MA is substantial, as approximately 30%–40% of all severe exacerbations occur in this population [2].

About two‐thirds of children and adolescents with asthma have intermittent or mild symptoms. However, they may still experience a progressive decline in lung function and a reduced quality of life because of inadequate prevention or suboptimal treatment [3].

Most literature on MA is adult‐focused, and some aspects of pediatric MA are incomplete or poorly defined. The existence of different pediatric age groups further complicates efforts to standardize disease management. In children and adolescents, MA may be underdiagnosed and undertreated, which contributes to disease progression, or even overdiagnosed, with unnecessary prescriptions [4].

This narrative review aims to engage the pediatric pulmonology community by raising clinically relevant yet unresolved questions regarding MA in children and adolescents, ranging from prevalence and definition to its pathophysiology, associated risks, and treatment. Given the importance of supporting clinicians in enhancing asthma care, we synthesized recommendations and statements on pediatric MA from current guidelines and documents, supplementing them with selected insights from the literature not covered in these sources.

We reviewed the most recently published versions of asthma guidelines and reports also including pediatric asthma, namely: (a) the US National Heart, Lung, and Blood Institute (NHLBI) guidelines [5]; (b) the British Thoracic Society, National Institute for Health and Care Excellence, and Scottish Intercollegiate Guidelines Network [6]; and (c) the Global Initiative for Asthma (GINA) Report [7]. We also carried out an electronic keyword literature search for English articles published on pediatric MA (Supporting File S1).

Our goal is to identify key gaps and open questions on pediatric MA, in the hope that addressing them may support the development of age‐appropriate future management strategies.

## 2 How Common Is Pediatric Mild Asthma?

The observation that 50%–75% of asthmatics are mild [3, 7] is based on few adult surveys [8, 9]. Data on pediatric MA are even less. One telephone survey reported a higher prevalence in children than adults (54.1% vs. 37%) [9], but this remains an isolated finding. In epidemiological studies, information about children is obtained by proxy, which may be less reliable than objective parameters, despite parents generally having a good perception of their child's asthma control.

The lack of robust epidemiological data in the pediatric population substantially limits our understanding of MA and its clinical characteristics. Therefore, there is a need to promote: (a) further epidemiological research to identify risk factors, monitor disease trends, assess healthcare resource needs, and inform effective prevention and management strategies for children and adolescents with MA; and (b) improved methodological approaches to better define its prevalence in the pediatric age, including clarification of whether data are reported by physicians, patients, or parents.

## 3 How Can Mild Asthma Be Defined in the Pediatric Age?

Accurately assessing pediatric MA requires a consistent definition that incorporates both physician and caregiver perspectives [10]. This includes understanding how the term “mild asthma” is interpreted, the context in which it is applied, and the criteria used to define it.

Defining pediatric MA remains indeed challenging due to the clinical variability of the condition [11]. Symptoms can range from minimal or absent to more frequent, but not daily manifestations, with no or rare episodes of exacerbation [7]. Diagnosis is typically made by pediatricians based on clinical examination, history, and, when feasible, spirometry. Parents often report symptoms and the associated triggers, but their description of severity may be discrepant from the report by the pediatrician, with overestimation of MA despite evident inadequate control [12].

Since the most significant events in MA are exacerbations, it is reasonable to question whether the term “mild” is appropriately understood in discussions with parents. Parents and patients should be adequately informed about the risk of asthma attacks; however, they may underestimate such warnings if the disease appears irrelevant to daily activities [10].

International guidelines and reports vary significantly in how MA is defined (Table 1). The previous US NHLBI guidelines classification between intermittent and mild persistent asthma based on symptom frequency was revised in 2020, and intermittent asthma (Step 1) was defined by symptoms less than twice/week and no exacerbations, while persistent asthma (Step 2) included symptoms on more than 2 days/week and up to 2 exacerbations/year [5].

**Table 1:** Current definition of mild asthma.

Source	Definition
US NHLBI [5]	Disease with symptoms less than twice a week and no exacerbations per year (Step 1)
GINA [7]	Disease that is well controlled with low‐intensity treatment, i.e., low‐dose ICS‐formoterol or low‐dose ICS plus as‐needed SABA (Step 1) The term “apparently mild asthma” or “asthma that seems to be mild” is preferred to the term *mild asthma*
ATS [13]	Disease with minimal symptoms and low risk in patients receiving (a) no pharmacological treatment or (b) SABA alone or (c) daily ICS plus SABA or (d) as‐needed ICS combined with SABA or formoterol Diagnosis based on retrospective clinical and functional parameters: – daytime symptoms < 2/week (impairment domain); – nighttime symptoms < 1/month (impairment domain); – fewer < 1 exacerbation/year (e.g., 1 every 2–3 years) (risk domain); – preserved lung function (postbronchodilator FEV_1_ > lower limit of normal) (risk domain)

Abbreviations: ATS, American Thoracic Society; FEV_1_, forced expiratory volume in 1 s; GINA, Global Initiative for Asthma; ICS, inhaled corticosteroids; SABA, short‐acting β2 agonist; US NHLBI, US National Heart, Lung, and Blood Institute guidelines.

Since 2019, the GINA report, which focuses on the treatment level required to achieve clinical control rather than symptom frequency alone, emphasized the risk of exacerbation even with intermittent symptoms, and advised against using short‐acting β_2_‐agonists (SABA) alone [14]. GINA defines MA as disease well controlled with low‐intensity therapy, such as as‐needed low‐dose inhaled corticosteroids (ICS) plus as‐needed SABA or as‐needed low‐dose ICS‐formoterol [7].

Recently, GINA experts have advised against using the term MA alone, as this label is often erroneously associated with a patient profile presumed to be at low risk of exacerbations and who does not need ICS‐containing treatment [7]. Alternatively, GINA experts have recommended using the term “apparently mild asthma” or “asthma that seems to be mild.” This distinction can be clinically useful in emphasizing the potential mismatch between symptom severity and underlying risks.

In 2023, the American Thoracic Society (ATS) published a consensus statement on MA, including pediatric considerations [13]. The ATS document defines MA as minimal symptoms and low risk in patients treated with no medications, or SABA‐only, or daily ICS plus SABA or as‐needed ICS combined with SABA or formoterol. Diagnostic criteria include daytime symptoms < 2/week, nighttime symptoms < 1/month, < 1/exacerbation/year, and preserved lung function (e.g., post‐bronchodilator Forced Expiratory Volume in 1 s (FEV_1_) > lower limit of normal). Despite these efforts, applying ATS definitions to children remains problematic. Poor symptom awareness and underreporting are common among patients and parents [10]. Spirometry is often unfeasible in younger children, and the bronchodilator threshold differs from adults [15]. Furthermore, pediatric asthma includes diverse age groups—preschoolers, school‐aged children, and adolescents—each with distinct clinical features and treatment responses [16].

A systematic review identified a list of common approaches used to define MA, based on criteria such as treatment level, symptom frequency, FEV_1_ thresholds, and specific definitions for children under age 5 [17]. This reflects the lack of standardization and complicates comparison across studies.

Ultimately, whether the definition for MA by current guidelines, reports and literature can be reliably applied to pediatric populations remains an open question. GINA and ATS criteria are retrospective and assume prior control, limiting their applicability to treatment‐naïve populations. Prospective longitudinal studies involving diverse cohorts of children and adolescents are needed to refine definitions and identify risk profiles. These would support the development of personalized treatment approaches and pediatric‐specific guidelines.

## 4 What Are the Main Pathophysiological Hallmarks of Pediatric Mild Asthma, and Do They Help to Determine Patients' Phenotypes and Endotypes?

Childhood asthma endotypes and phenotypes have been widely described [18, 19, 20, 21]. A latent class analysis of 9–15‐year‐old twins identified the MA phenotype in 9% of patients, who had fewer symptoms and exacerbations but still required preventers in over half of cases [22]. Similarly, a recent UK clustering study found a “mild transient wheeze” phenotype in about 17% of children, most infection‐related and resolving at ages 5–8 [23].

Despite their milder clinical symptoms, patients with MA have historically exhibited airway inflammation [24]. Data on airway inflammation in pediatric MA is limited compared to adults' studies. School‐aged children with intermittent symptoms often show eosinophilic inflammation, albeit milder than in severe cases [25, 26]. In adults, mild symptoms can lead to airway remodeling and irreversible structural changes [27], underscoring the importance of early anti‐inflammatory treatment [28, 29]. Few histological studies have investigated this in children, largely due to ethical restrictions on invasive procedures, which can be substituted by other noninvasive biomarkers for endotype identification [30, 31, 32].

In conclusion, our understanding of the inflammatory endotypes and underlying pathophysiological mechanisms of pediatric MA remains limited. Future research should focus on identifying and validating noninvasive biomarkers (e.g., on blood, urine, and/or induced sputum) in diverse age groups to characterize the inflammatory profiles associated with disease activity and the at‐risk phenotypes, ultimately supporting a more personalized approach to treatment.

Despite the paucity of published studies, clarifying phenotypes and endotypes in pediatric MA is critical to improve tailored treatment approaches and to better understand the pathophysiology of the condition in diverse age groups.

## 5 Do Children With Mild Asthma Face Significant Risks? If so, What Is the Long‐Term Prognosis of Pediatric Mild Asthma?

The primary goal of asthma management is to maintain long‐term control and minimize future risk, particularly by preventing exacerbation, a key marker of asthma severity at any age [7, 33]. This is particularly important in patients with MA in whom the rate of exacerbations can be as high as 2.88 events/year, and 30%–40% of these are severe [17].

In pediatric populations, even those classified as mild may be at risk. A trial of the leukotriene receptor antagonist (LTRA) montelukast in children aged 2–14 years with intermittent asthma reported a 2.9% hospitalization rate due to exacerbation [34]. However, accurately identifying children with intermittent asthma who are at low or high risk for severe outcomes remains a challenge [35]. Misperception of symptoms and limited access to spirometry can obscure severity [36], and fatal asthma attacks have been reported in adolescents with mild symptoms not undergoing regular medical follow‐up in the year before death [37].

**Figure 1:**
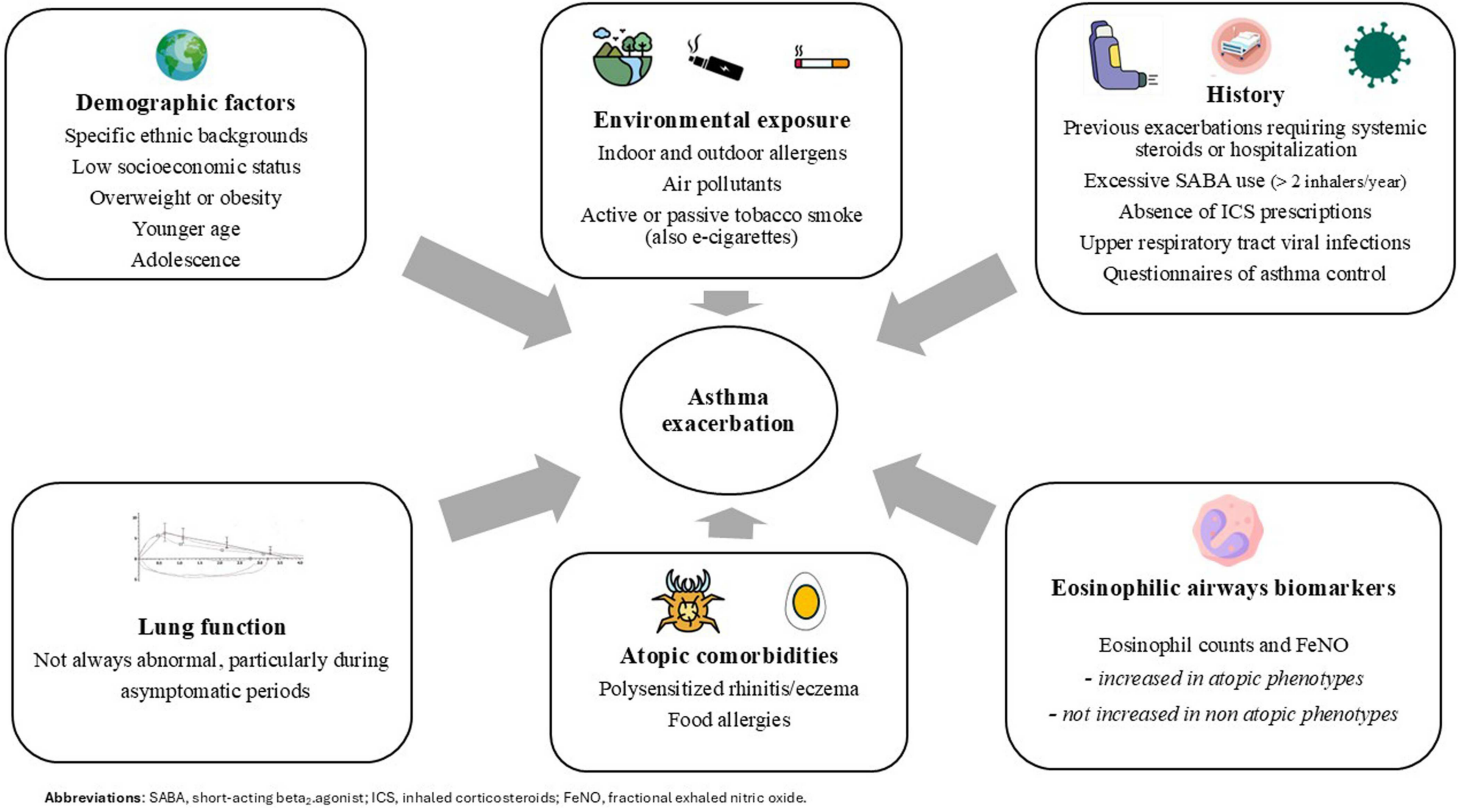
Schematic presentation of the predictors of asthma exacerbations in children and adolescents with mild asthma. [Color figure can be viewed at wileyonlinelibrary.com]

Although predictors of exacerbation in pediatric asthma have been extensively studied, specific data in children with MA are limited. The predictors of exacerbation reported in pediatric MA studies (Figure 1) include demographic variables [38, 39], as well as exposure to allergens, pollutants, and tobacco smoke—although the latter associations are weaker in MA compared to severe forms [40]. Importantly, poor outcomes can be strongly predicted by a history of exacerbations requiring systemic corticosteroids and overuse of SABA (i.e., > 2 inhalers/year) without ICS [35, 38, 41, 42, 43]. Upper respiratory infections may act indeed as early warning signs for deterioration, even in seemingly well‐controlled children [44]. Polysensitized children with allergic rhinitis, eczema, or food allergies with mild symptoms should be monitored due to high exacerbation risk [45]. In one study of intermittent asthma, atopic children showed higher ICS levels and blood eosinophils than nonatopic peers, though differences were not statistically significant [26]. Spirometry can help assess control, but many children with mild symptoms present with normal lung function, both at baseline and during follow‐up [46]. Despite these challenges, noninvasive biomarkers such as fractional exhaled nitric oxide (FeNO), blood or induced sputum eosinophils, allergen sensitization, and lung function may help stratify risk, and even normal findings may warrant repeat assessment in follow‐up. Finally, tools like the Asthma Control Questionnaire and Asthma Control Test (ACT) offer additional insights into previous symptom patterns and deterioration risk [47, 48]. However, these tools may be limited by inconsistent responses or incomplete data. Relying solely on subjective assessments, such as the ACT's “How has your asthma been over the past 4 weeks?”, without considering past exacerbations, may underestimate the risk of future attacks [49].

The long‐term prognosis of MA, especially when starting in childhood, is generally favorable [3]. However, disease progression is variable. In one longitudinal study, 8% of 14–45‐year‐old patients with newly diagnosed MA progressed to more severe forms over 10 years [50]. Risk factors included older age at onset and suboptimal use of SABA without ICS in the 1st year. To date, no equivalent studies have investigated this trajectory in younger children with MA. Recent longitudinal lung function data indicate that FEV_1_ rises rapidly in early life, peaks in early adulthood, and then declines [51], supporting monitoring even in patients with intermittent symptoms.

In pediatric MA, identifying reliable, accessible, and noninvasive biomarkers is essential for improving patient phenotyping and predicting disease progression. However, literature on risk biomarkers is notably lacking in young children, an age group in which preventing exacerbations is crucial to limiting disease progression toward moderate‐to‐severe forms. Finally, while the overall outlook is reassuring, ongoing clinical monitoring is imperative. It enables early intervention, prevents deterioration, and supports the implementation of personalized, disease‐modifying strategies.

In conclusion, as there is currently no universally accepted strategy regarding the optimal timing and modalities for monitoring children and adolescents with MA, particularly those at risk of life‐threatening exacerbations, more research is urgently needed. Future studies should also evaluate the role of innovative approaches, including those based on digital health technologies, as well as established home‐based monitoring tools, such as peak expiratory flow and FeNO, with the aim of identifying reliable predictors of disease deterioration and improving risk stratification.

## 6 What Is the Best Therapeutic Strategy for Children and Adolescents With Mild Asthma?

At any age, exposure to respiratory viruses and allergens drives airway inflammation that deserves treatment [4]. In recent years, evidence has accumulated showing that MA should no longer be treated with SABA alone [52]. Regular SABA use is associated with reduced bronchodilator response, worsened eosinophilic inflammation, and increased mortality [53]. In contrast, ICS, the cornerstone of asthma treatment, reduces inflammation, improves bronchodilator responsiveness via β_2_‐receptor upregulation, and lowers the risk of severe exacerbations [54]. Adherence to ICS remains poor indeed, especially in children and adolescents, often due to concerns about growth [55].

In children with intermittent symptoms, both undertreatment with SABA‐only and overtreatment with systemic corticosteroids carry substantial risks [56]. We reviewed the latest evidence to guide age‐specific therapeutic strategies. We presented recommendations for three distinct groups: preschool children (< 5–6 years), school‐aged children (6–11 years), and adolescents (> 11 years), based on current guidelines and reports plus selected recent studies not included in these sources in pediatric MA patients.

### 6.1 Children < 5–6 Years Old

Management of asthma in young children with intermittent symptoms remains complex due to variable phenotypic heterogeneity and different responses to asthma medications [57, 58]. According to GINA 2026, children under 5–6 years with infrequent wheezing (Step 1) should preferably receive as‐needed SABA monotherapy, but due to concerns about SABA overuse, GINA recommends adding short courses of ICS at the onset of respiratory infections, especially in atopic children, without daily maintenance therapy. Intermittent high‐dose ICS should be used only when proper technique and adherence can be ensured. GINA also strongly recommends using an inhaler for treatment and further emphasizes how assessing whether the inhaler is being used correctly reduces the risk of exacerbation [7].

Similarly, the NHLBI 2020 guidelines recommend as‐needed SABA, with a short daily ICS course during respiratory infections in children with intermittent symptoms [5]. However, neither GINA nor NHLBI specify the duration of “short” ICS courses. Given that post‐viral lower airway symptoms are usually resolved within 10 days [59], intermittent ICS should likely not exceed this timeframe. The NHLBI recommends monitoring growth in children on repeated ICS courses.

In contrast, in children under 5 the BTS/NICE/SIGN guidelines support daily ICS (with or without SABA) over intermittent ICS, showing better outcomes in reducing exacerbation and hospitalization [6]. However, the guidelines do not provide tailored guidance for children with strictly intermittent symptoms.

Two additional therapeutic options in children aged < 5–6 years warrant discussion, that is, the LTRA montelukast and the combination of ICS with the fast‐acting β_2_‐agonist formoterol. No current guidelines support LTRA in intermittent asthma at this age, due to insufficient evidence [5, 6, 7]. While promising in older children with uncontrolled asthma [60, 61], ICS/formoterol combination, namely budesonide/formoterol either as as‐needed‐only anti‐inflammatory relief (AIR‐only) therapy for managing symptoms or as maintenance and reliever therapy (MART) is not approved in most countries in children < 5 years old and is not recommended by NHLBI and BTS/NICE/SIGN guidelines and by GINA report [5, 6, 7], but ongoing pediatric studies offer hope at this age too.

In conclusion, more studies are needed to compare as‐needed ICS versus regular ICS in preschoolers with intermittent symptoms.

### 6.2 Children 6–11 Years Old

Managing asthma in children aged 6–11 may be difficult because of developmental changes, including increasing autonomy, evolving adherence patterns, and variable symptom perception. Low adherence and poor understanding of treatment plans are common, especially when symptoms are minimal or not daily [10]. These factors must guide treatment choices in this age group.

GINA recommends that children of this age group with intermittent symptoms take ICS whenever SABA is used, instead of SABA monotherapy, even in well‐controlled cases [7]. This reflects increasing concern over SABA overuse and undertreatment of airway inflammation by MA patients [52, 53].

In children with asthma symptoms occurring twice a week or less (Step 1) the GINA 2026 report recommends, as the preferred approach, the AIR‐only strategy. This includes two options: as‐needed low‐dose ICS–formoterol or as‐needed low‐dose ICS–SABA (administered as a combination or in separate inhalers), without daily maintenance therapy [7].

In contrast, NHLBI 2020 continues to recommend SABA alone for children 5–11 years old with intermittent asthma, citing insufficient evidence supporting intermittent ICS [5]. The Panel based the recommendation on a trial of 5–18‐year‐old children with mild *persistent* asthma previously on controller therapy [62], limiting its relevance to truly intermittent cases. Additionally, results were not stratified for the 6–11 age group.

The BTS/NICE/SIGN guidelines recommend daily low‐dose ICS over intermittent ICS in children aged 5–11, primarily to reduce exacerbation risk [6]. This recommendation has been recently supported also by a meta‐analysis comparing regular versus as‐needed ICS in school‐aged children with MA, that found regular ICS had a slightly greater, though not significant, reduction in nonsevere exacerbations [RR 0.83 (95% CI: 0.61–1.12)] [63]. However, this analysis included only three studies [61, 63, 64], two of which enrolled children with mild persistent asthma and did not provide stratified data for intermittent symptoms, limiting the generalizability [62, 65]. Importantly, as‐needed ICS outperformed LTRA, reinforcing ICS as the preferred option [66].

The MART regimen (ICS/formoterol in single inhaler) is not licensed in most countries for use in 6–11‐year‐olds with intermittent asthma [67], and both GINA and BTS/NICE/SIGN restrict MART to more severe stages (Steps 3–4) [6, 7]. The NHLBI 2020 experts conclude that trials for Step 1 patients in this age range are ongoing [5]. A recent open‐label randomized controlled trial of 2 inhalations of budesonide–formoterol 40/2.25 versus 2 inhalations of salbutamol 100 mcg as reliever monotherapy in children 5–15‐year‐old with MA using their SABA reliever 2·2 times/week showed that switching to as‐needed budesonide–formoterol reduced the rate of asthma attacks by 45% compared with as‐needed SABA [RR 0.55 (95% CI 0.35–0.86)] [68]. In this study, patients aged 12–15 years (72%) had indeed a medication effect size larger than 5–11‐year‐old children (35%), suggesting potentially greater efficacy of budesonide–formoterol at preventing asthma attacks in older subjects. Limitations of this study include the open‐label design (even though this increased the external validity of the findings of the real‐world practice), the absence of dose counters and the fact that the study was conducted during the COVID‐19 pandemic, when stringent public health measures and fewer circulating respiratory viruses likely contributed to lower‐than‐predicted rate of severe asthma attacks.

In conclusion, further studies are needed to define the optimal therapeutic strategy for children younger than 12 years with MA. Well‐designed clinical studies should be promoted to evaluate the efficacy and long‐term safety of the AIR‐only strategy to generate the evidence required for local regulatory approval and to support its implementation in clinical practice.

### 6.3 Adolescents (> 11 Years Old)

Adolescents with MA face unique challenges, including frequent misperception of symptoms and low adherence, often influenced by peer comparison and self‐image concerns [66]. These factors can lead to suboptimal asthma control.

GINA offers two therapeutic tracks for adolescents with intermittent symptoms [7]. In Track 1 (preferred), as‐needed low‐dose ICS/formoterol is recommended for symptom relief as AIR‐only therapy, without any maintenance therapy. Evidence from trials involving adolescents shows that this approach reduces severe exacerbations by up to 65% compared to SABA‐only, with similar symptom control and fewer hospitalizations [69, 70]. It is also cost‐effective and easy to use in a single inhaler [71]. However, in some countries (including those of the European Union), this combination is not licensed for adolescents, raising issues with off‐label prescribing, or may not be accessible in low‐income settings [72]. In Track 2 (alternative), if Track 1 is unavailable or if the patient is stable and adherent, as‐needed low‐dose ICS alongside SABA is recommended. ICS should be taken either in a unique inhaler or separately soon after SABA. A recent trial has suggested that ICS/SABA in unique inhaler reduces exacerbation risk by 53% compared to SABA‐only, though results may not apply to well‐controlled adolescents due to low representation of this age group [73].

In contrast, NHLBI 2020 recommends SABA‐only for adolescents with intermittent symptoms [5], while BTS/NICE/SIGN recommends as‐needed ICS/formoterol in those ≥ 12 years old, provided licensed inhalers are used [6]. Recently, a meta‐analysis found that both as‐needed ICS/formoterol [RR 0.73] and regular ICS/long‐acting β_2_‐agonists [RR 0.68] are more effective than regular ICS alone in preventing severe exacerbations in adolescents with MA [63].

An overview of the treatment of pediatric MA, as outlined in current international guidelines and reports, reveals that some recommended strategies for children and adolescents are not fully aligned (Table 2). This lack of consistency might generate doubts among pediatricians, resulting in sometimes hazy information for patients and their families, and contributing to suboptimal management.

**Table 2:** Summary of recommended therapeutic strategies for the management of mild asthma in different pediatric age groups.

Source	Children < 5–6 years old	Children 6–11 years old	Adolescents (> 11 years old)
US NHLBI [5]	As‐needed SABA + ICS short daily course at onset of respiratory infections	As‐needed SABA	As‐needed SABA
BTS/NICE/SIGN [6]	Daily ICS alone or in combination with SABA	Daily low‐dose ICS	As‐needed low‐dose ICS‐formoterol
GINA [7]	As‐needed SABA (*preferred*) Intermittent ICS short course at onset of respiratory infections, whenever SABA is taken, especially if underlying atopy (*alternative*) *without maintenance therapy*	Preferred AIR‐only options:	*Track 1*: as‐needed low‐dose ICS‐formoterol as AIR‐only therapy, *without maintenance therapy*
		– As‐needed low‐dose ICS‐formoterol	
		OR	*Track 2*: as‐needed low‐dose ICS whenever SABA is taken
		– As‐needed low‐dose ICS‐SABA (combined or separate inhaler) *without maintenance therapy*	

Abbreviations: AIR, anti‐inflammatory reliever; BTS/NICE/SIGN, British Thoracic Society/National Institute for Health and Care Excellence/Scottish Intercollegiate Guidelines Network; GINA, Global Initiative for Asthma; ICS, inhaled corticosteroids; SABA, short‐acting β2 agonist; US NHLBI, US National Heart, Lung, and Blood Institute guidelines.

Whether and how to treat children and adolescents with intermittent symptoms is a topic of current debate [63]. Most published trials include adolescents, and there are less studies specifically involving children under 12 years of age—for example, comparing daily ICS use to as‐needed ICS combined with bronchodilators in a single inhaler—although findings in adolescents have been encouraging [53, 69, 70, 73, 74]. Until the uncertainties around treatment are addressed, and the underlying airway inflammation not undertreated, the burden of MA will remain. A more standardized approach would be ideal, but it must not overlook individual preferences as well as differences in medication, inhaler costs, and local regulatory approvals [74]. These challenges can be addressed by adopting flexible regimens that consider the patient's age, the needs of both the patient and the family, and the prescribing options available in the country of residence [75].

## 7 Conclusions

In recent years, our understanding of MA has advanced considerably, however, its management in childhood remains incomplete, at least for some aspects. Despite being labeled as “mild,” this phenotype can still result in significant morbidity, particularly due to exacerbations that are often underestimated or poorly managed. Therefore, improving outcomes in the novel defined “apparently mild asthma” must be considered a priority for the pediatric population. To this end, further prospective, longitudinal studies are urgently needed, particularly those designed to clarify natural history, phenotypes, and endotypes across different pediatric age groups.

Ultimately, important unmet needs remain in the management of children and adolescents with MA. Addressing these knowledge gaps should be a priority for future pediatric asthma research to optimize disease prevention, monitoring, and treatment (Table 3).

**Table 3:** Unmet needs and future directions in pediatric mild asthma research.

	Unmet needs	Future directions
Pathophysiology	Limited knowledge of the inflammatory endotypes underlying pediatric mild asthma and the pathophysiological mechanism.	To investigate noninvasive biomarkers (blood and/or urine and/or induced sputum) to identify the inflammatory profiles associated with disease activity (at‐risk phenotypes) and to address treatment.
Monitoring	No universally accepted strategy defining the optimal timing and modalities for monitoring children and adolescents with mild asthma, particularly those at risk of life‐threatening asthma attacks.	To promote studies on the role of digital health and home‐based peak flow/FeNO monitoring aimed at identifying reliable predictors of disease deterioration.
Treatment	Limited use of AIR‐only therapy in children and adolescents because	To promote studies on efficacy and long‐term safety of AIR‐only in children under 12 years, particularly in younger children, to generate the evidence needed to support approval by regulatory authorities and implement the clinical practice.
	– as‐needed‐low‐dose ICS–formoterol not approved in many countries	
	– as‐needed ICS plus SABA in a single inhaler not widely available and not sufficiently evaluated across all pediatric age groups.	
Implementation	Low adherence in as‐needed ICS regimens	To eliminate barriers to low adherence to as‐needed ICS regimens by increasing awareness of ICS clinical benefits, reinforcing the safety profile or minimal risk of adverse effects, and bearing in mind patients' and parents'/caregivers' preferences.
	Lack of consistency among current guidelines and reports with hazy information for patients/families, and suboptimal management.	To foster international consensus among experts on a shared algorithm for the management of mild pediatric asthma that takes into account the prescribing options and regulatory approvals in each country.

Abbreviations: AIR, anti‐inflammatory reliever; FeNO, fractional exhaled nitric oxide; ICS, inhaled corticosteroids.

Healthcare providers caring for children and adolescents with MA must work toward a standardized diagnostic approach and deliver tailored therapeutic plans that reflect the needs of individual patients and their families. Greater effort should be made to translate scientific advances into best clinical practice, particularly through multidisciplinary collaboration among clinicians, researchers, and patients' communities. Clinical trials of anti‐asthma therapies currently approved for adults should be expanded to include children with intermittent or mild symptoms. Also, regulatory authorities should be encouraged to consider label extensions for achieving broader drugs accessibility. Special attention must be paid to children living in low‐income countries, or in socioeconomically disadvantaged settings, who are often excluded from research. Future studies should prioritize their inclusion and address the high cost of asthma medications, which remains a major obstacle to treatment access worldwide [76].

In conclusion, future research efforts should aim to (a) establish a standardized and widely accepted definition of the MA phenotype in children; (b) identify risk factors and early predictors of poor outcomes, even in “mild” or “apparently mild” asthma pediatric cases; (c) evaluate pharmacological strategies through well‐designed trials to support personalized therapy in both younger and older pediatric patients. Based on the evidence reviewed in this article, it is both timely and necessary to address these gaps in knowledge and clinical practice to ensure better care and long‐term outcomes for all children with MA, through an algorithm ideally shared by multiple experts from different countries.

## Author Contributions

**Francesca Santamaria:** conceptualization, methodology, writing – review and editing, writing – original draft, project administration, resources, investigation, funding acquisition. **Cristina Fontanella:** investigation, writing – review and editing, data curation, funding acquisition, visualization, methodology, validation. **Melissa Borrelli:** visualization, writing – review and editing, investigation, resources, supervision, formal analysis, project administration.

## Funding

The authors have nothing to report.

## Conflicts of Interest

The authors declare no conflicts of interest.

## Supporting information


Supporting File

## Data Availability

The data that support the findings of this study are openly available in PubMed at https://pubmed.ncbi.nlm.nih.gov/.
