# Supplementary material for: Pediatric Mild Asthma: Bridging Current Gaps to Improve Future Care
Source: Pediatr Pulmonol. 2026 Aug 21;61(8):e71800. doi: 10.1002/ppul.71800 (PMC13494689; doi:10.1002/ppul.71800)
Supplement: Supplementary file 1 [file PPUL-61-0-s001.docx]

**Supplementary file**

We carried out an electronic keyword literature search for English articles published on pediatric MA since January 1, 2000, up to February 25, 2026, in the Scopus, Web of Science, PubMed, and MEDLINE databases. We excluded studies conducted exclusively on adults but included those with a population comprising children (or adolescents) and adults. The terms “mild asthma” AND (intermittent OR pediatric OR children OR preschool children OR adolescent OR asthma guidelines) were used as keywords in combination, and studies found were evaluated for selecting relevant literature. In addition, a manual search was conducted to evaluate review articles’ references.
